# Q and A with Outgoing ASTMH President Chandy John

**DOI:** 10.4269/ajtmh.19-Interview

**Published:** 2019-11-20

**Affiliations:** Chandy C. John, MD, MS, FASTMH, recently completed his term as ASTMH President. He sat down with science writer Matthew Davis to reflect on his work with the society and an active year in global health that saw members closely involved in a number of topical issues, including the Ebola outbreak, issues of immigrant health, and access to malaria medications for patients in the United States. Dr. John holds the Ryan White Endowed Chair in Pediatric Infectious Diseases and is the director of the Ryan White Center for Pediatric Infectious Diseases and Global Health at the Indiana University School of Medicine.

## Looking back on your year as President, what stood out for you—within the society itself, in the world of global health and tropical infectious diseases, and in the broader public conversations that impact the work of your members?

There are a several things that stand out.

First, I have a much greater appreciation for the dedication, passion, and hard work of our members. When you serve as president you see how valuable it is to have access to the thoughtful perspectives from such a broad group, from trainees to people who have been with the society for 50 years. We have so many people who care very deeply about the work we do and are committed to our future. The fact that they are willing to serve on committees and run task forces and teach courses in addition to their day jobs is very impressive.

**Figure:**
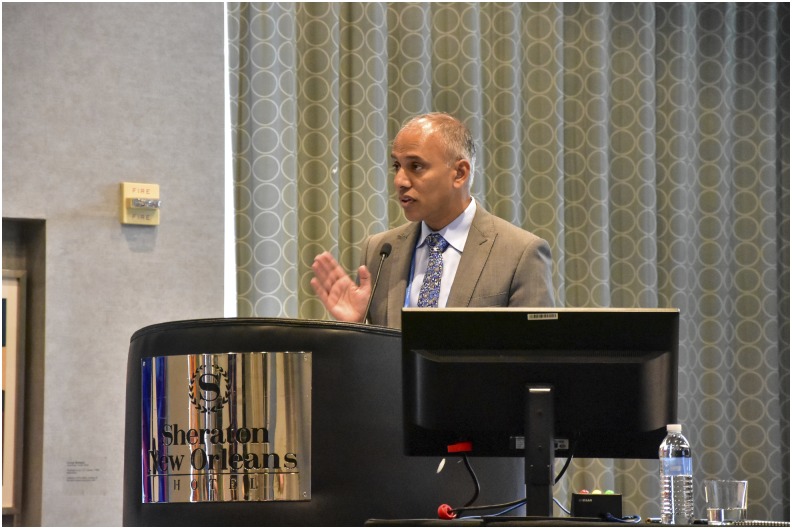
President Chandy C. John, MD, MS, FASTMH, addresses the audience during the Society’s business meeting at the 2018 Annual Meeting in New Orleans, Louisiana, shortly after taking office.

Second, I have a new appreciation for the value of a knowledgeable CEO like Karen Goraleski and the team. They are just as engaged as our membership, and they are working everyday with us to implement our vision. They know the content, and equally important, they believe in our mission of making the world a better place by getting rid of tropical and infectious diseases.

Third, I have learned the value of a strategic plan. It can seem like a slog, but without refreshing our strategy every three years, we run the risk of doing the same things, while the world has moved on.

Looking at the world of global health, the last year demonstrated the need to stay focused on long-term goals while also being able to address more immediate issues.

We need to keep working to eradicate diseases like malaria and onchocerciasis. But, then, there are issues that arise like the Ebola outbreak, immigrants not getting influenza immunizations, or the issues over measles vaccination. The need to address what’s happening in the moment while balancing that with attention to long-term priorities is an art—and managing that balance was a real revelation to me. I also appreciate the need to consider big picture issues that don’t fit neatly into a single category. For example, how do we engage climate experts to understand the many different ways it affects the diseases we care about?

Finally, I learned the importance of communications and how to engage with different stakeholders and audiences. For example, it’s important to engage people in the U.S. government, speak their language, and advocate for our interests in a diplomatic way that does not come across as brash or condescending.

## The society recently made a very strong statement about withholding seasonal influenza vaccinations from migrants at border detention facilities. How has the society changed and how and when it uses its voice?

I think the society is learning more and more about the importance of making our voice heard in areas where we have expertise. When it comes to immigrant and migrant health, we have people who are experts in this area, and it was clear to them that withholding influenza vaccinations from migrants in detention facilities was a very bad idea. It was not good for the health of migrants or for others. But when we decided to bring our voice to this issue, it was not simple to enter the fray. It was because we had a fact-based statement to contribute, backed by a lot of evidence and experience. It would have been irresponsible to stay silent when we knew that what was happening was not good policy and not good for public health. And again, I credit Karen with helping us understand the importance of advocacy and identifying key moments when it is appropriate and necessary to use our voice.

## The society also reached out to FDA and CDC this year about making artesunate more widely available for treating malaria patients seeking care in the United States. Why does this matter?

Artesunate is the gold standard for treating severe malaria, but it’s easier to get in Uganda and Kenya than in the United States. Yet we do have about 300 severe malaria cases in the United States every year. As a physician at Riley Hospital for Children in Indianapolis, I do take care of children with severe malaria. We currently have an extraordinary situation where there is no FDA-approved treatment for malaria in the United States, and to get artesunate, you have to obtain it from the CDC, which can result in unacceptable delays for the treatment of a very sick patient. I think it’s an appropriate role for ASTMH to bring all of the key partners together, communicate the complexity of the situation, and look for a better solution. We are doing that, and I hope there will soon be an FDA-approved form of artesunate available for hospitals to have on their formulary. In the meantime, we appreciate the CDC’s role in making sure artesunate is available for patients who need it. The current solution is not optimal, but at least it does ensure there is a way to get artesunate in the United States.

## What do you think the global response to the Ebola outbreak in DRC tells us about the state of support for global health priorities? It is a unique situation, given the extraordinary challenges in particularly unstable parts of the country, but are there lessons to be learned here?

I think, the biggest thing we have learned is that there are no simple answers to this crisis. There are so many things going on, including people believing the disease doesn’t even exist, the murder of health care workers, the understandable suspicion in local areas with Ebola of outsiders coming to “fix” the situation, resistance to using vaccines, and the deliberations over whether to designate the outbreak a public health emergency of international concern. What’s important is to maintain interest and advocacy efforts after the news cycle has tailed off. We saw this after the West Africa outbreak in 2014. Ebola was seen as a huge problem, then the interest died down. But the disease did not go away.

## When we spoke last year, you talked about a desire to increase involvement from international members, increase retention of trainee members, and advocate for the sub-specialty board certification in global health and tropical medicine. How are things going in each of these areas?

My natural inclination is to move rapidly toward a solution, but all of these areas are complicated and need thought and time to consider priorities and best approaches. The board did move ahead immediately with one initiative that makes a start on increased international and trainee member participation: the Presidents’ Challenge Travel Fellowship Fund.

When fully funded (and we are two-thirds of the way there), this fund will provide 100 additional travel awards to the ASTMH annual meeting over 5 years. We expect 75% of these awards will be to trainees from low- and middle-income (LMIC) countries, so the president’s challenge addresses LMIC and trainee members in a very practical way. And I do want to say that past (and future) presidents of ASTMH came through in a big way for this fund, as did board members and society members in general.

For more long-term solutions, we have formed specific committees on international members, trainee members, and tropical medicine and global health certification. I have had to rein in my desire to see all of this done yesterday because we need to carefully consider the best ways to move ahead. Each of the committees is developing objectives and key results for their areas to work on over the next 3 years, so that we can assess whether we have made progress.

We also just hired a development officer to assist with fundraising, as achieving our goals in all of these areas will require consistent funding. I’m confident that we will see substantial progress in the next 3 years.

